# Supplementary material for: Wafer-scale solution-processed 2D material analog resistive memory array for memory-based computing
Source: Nat Commun. 2022 Jun 1;13:3037. doi: 10.1038/s41467-022-30519-w (PMC9160094; doi:10.1038/s41467-022-30519-w)
Supplement: Supplementary file 1 — Supplementary Information [file 41467_2022_30519_MOESM1_ESM.pdf]

Supplementary Information

Wafer-scale Solution-Processed 2D Material Analog Resistive Memory Array for Memory-Based Computing

Baoshan Tang[1], Hasita Veluri[1], Yida Li[1], Zhi Gen Yu[2], Moaz Waqar[3], Jin Feng Leong[1], Maheswari Sivan[1], Evgeny Zamburg[1], Yong-Wei Zhang[2], John Wang[3], Aaron V-Y Thean[1]*

[1] *Department of Electrical and Computer Engineering, National University of Singapore, Singapore 117576, Singapore.*

[2] *Institute of High Performance Computing, Singapore 138632, Singapore.*

[3] *Department of Materials Science and Engineering, National University of Singapore, Singapore 117574, Singapore.*

**Corresponding author. E-mail: Aaron.Thean@nus.edu.sg*

Supplementary Figures

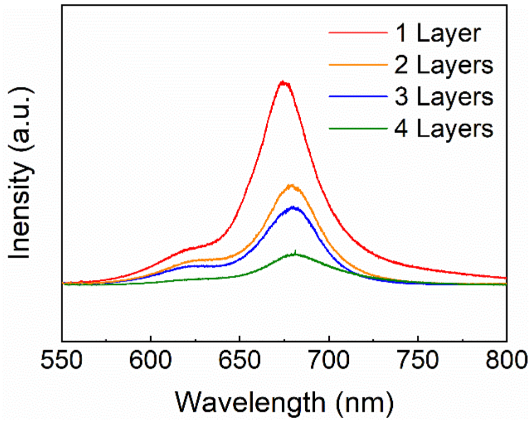

Supplementary Figure S1 | Photoluminescence (PL) Characterizations of the exfoliated MoS_2 nanosheets deposited on Si substrate. Strong PL peak at 678 nm in monolayer MoS_2 has been observed, together with reduced PL intensity when layer thickness increases. The PL results reveal the good quality of the exfoliated MoS_2 nanosheets.

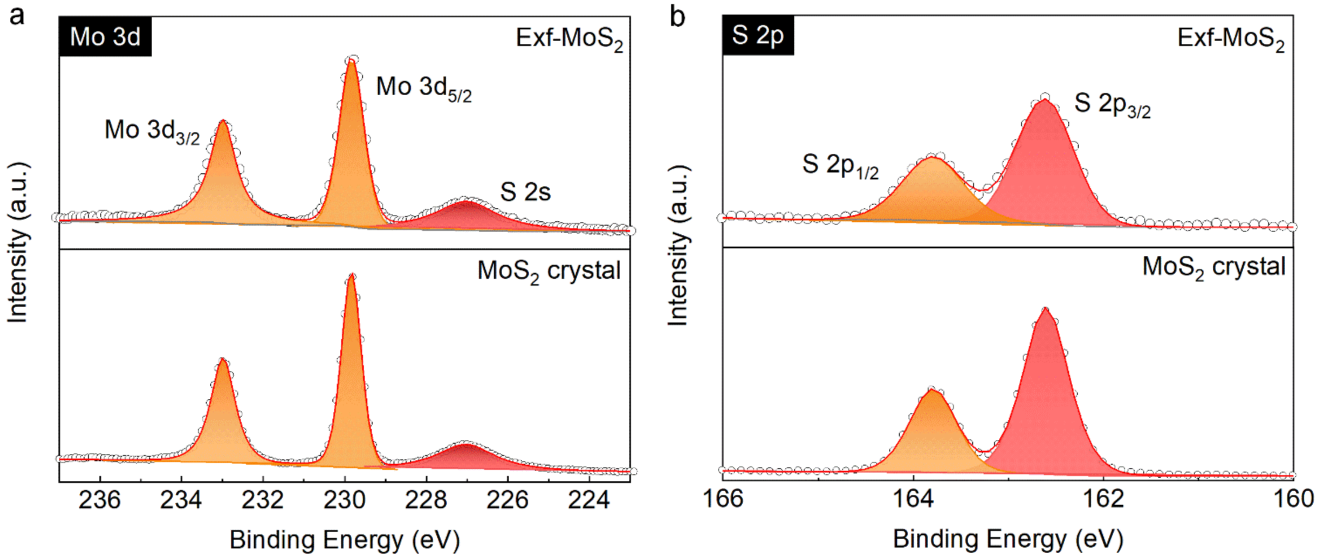

Supplementary Figure S2 | Investigation of surface chemistry and stoichiometry in liquid exfoliated MoS_2 nanosheets. MoS_2 single crystal was used for comparison. **a.** Mo 3d and **b.** S 2p region. In Mo 3d region, two

strong peaks at 229.5 and 232.6 eV are observed, corresponding to Mo^{4+} $3d_{5/2}$ and Mo^{4+} $3d_{3/2}$, respectively. The absence of additional peaks at higher binding energies in exf-MoS_2 (exfoliated MoS_2 nanosheets) suggests that the exf-MoS_2 nanosheets exhibit a pristine chemical state with no oxidation. From the integrated area of the XPS peaks, the stoichiometry of Mo and S ratio has been calculated to be around 1:1.93, indicating 3.5% sulfur vacancies (V_S) in liquid exfoliated MoS_2 nanosheets.

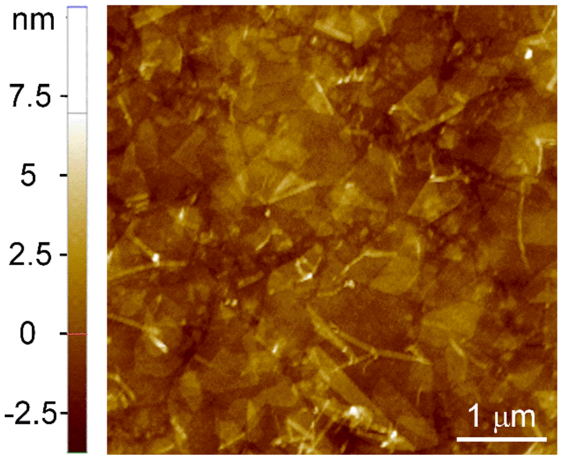

Supplementary Figure S3 | The AFM topographic images of the spin coated MoS_2 film. The surface has a low average roughness of 1.2 nm across a 5×5 μm area.

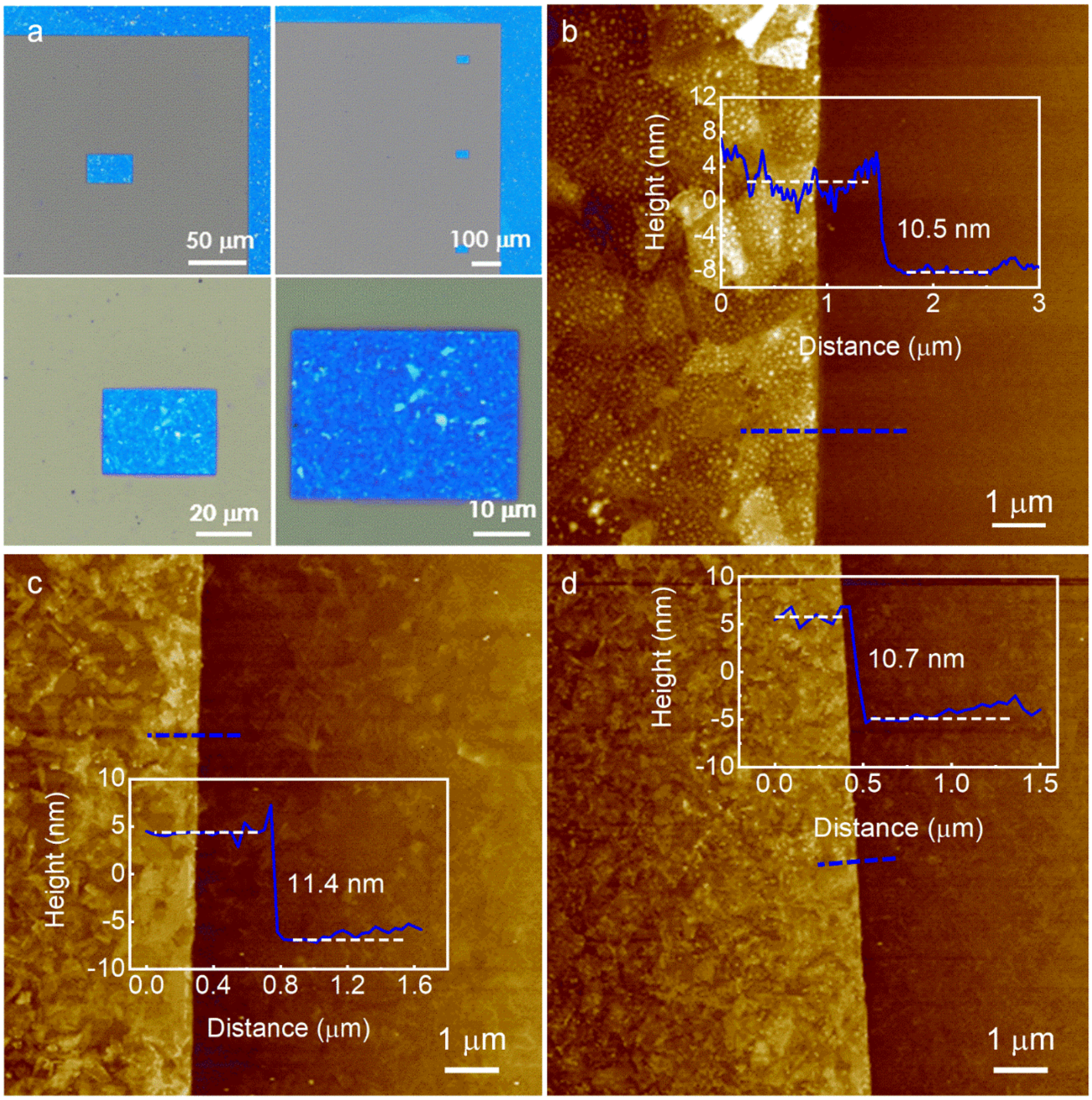

Supplementary Figure S4 | Optical image and corresponding AFM images of the spin coated MoS_2 film after patterning and etching. a. Optical images; **b-d.** AFM images of MoS_2 films from Suspension C, B and A, respectively. The inset is the step profile measured at the edge of the MoS_2 film. The square-shaped MoS_2 thin film was patterned and etched to measure the film thickness. Three types of MoS_2 suspensions result in similar thickness with excellent coverage.

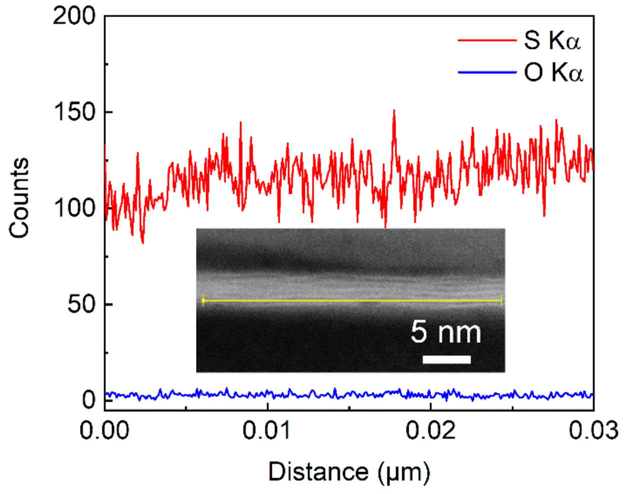

Supplementary Figure S5 | Cross-sectional elemental profile in MoS_2 switching layer. inset: cross-section high-angle annular dark-field (HAADF) image of MoS_2 switching layer. As compared to S, the weak O signal suggests that negligible oxygen atoms are present in MoS_2.

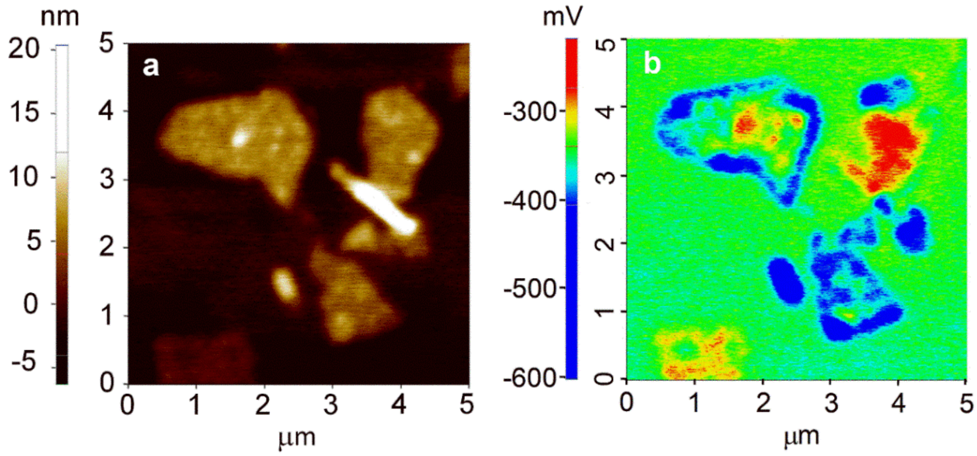

Supplementary Figure S6 | Kelvin probe force microscope (KPFM) measurement of the MoS_2 nanosheets on Pt support. a. AFM image of MoS_2 nanosheets and the corresponding KPFM (b). The edge region of the MoS_2 nanosheets tend to have more negative potentials as compared to the central part, implying more vacancies located at the edge region.

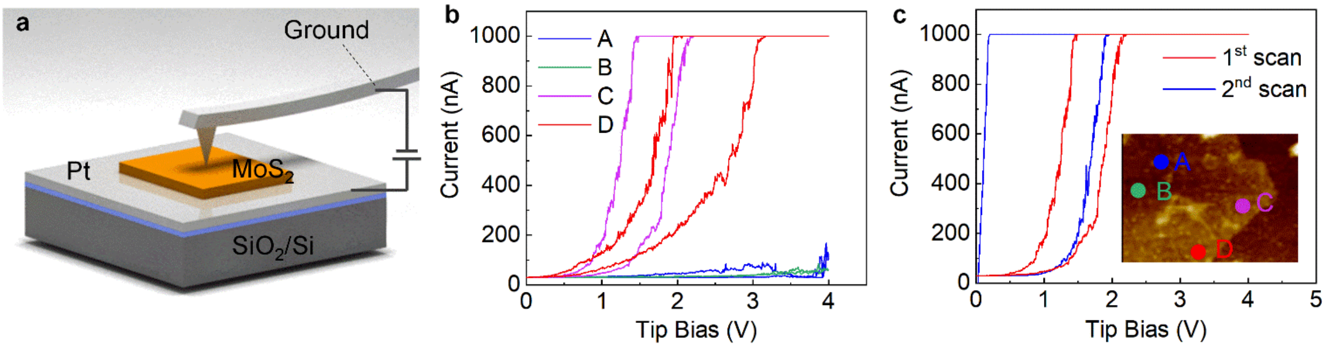

Supplementary Figure S7| Conductive atomic force microscope (C-AFM) characterization of the MoS_2 nanosheet. a. Schematic diagram of the C-AFM configuration. **b.** *I-V* characteristics measured by C-AFM at the edge (C, D) and the center (A, B) of MoS_2 nanosheet. **c.** Two repeated *I-V* characteristics measured at point C. As for C-AFM measurement, the MoS_2 nanosheets were drop cast onto clean Pt-coated Si wafer. Figure S7a shows the set-up for the C-AFM, in which the C-AFM tip coated with Pt was grounded while positive bias was applied to the Pt coating on Si wafer. The current is detected as a function of the applied bias. As shown in Figure. S7b, the MoS_2 edge shows strong resistive switch (RS) effect at voltage below 3 V. But the middle of the MoS_2 nanosheet shows near hysteresis-free *I-V* even at higher positive bias of 4 V. Repeated *I-V* sweep at point C shows the increase in the conductance of the MoS_2 nanosheets (Figure S7c), implying the formation of conductive filament. The C-AFM experimental results reveal that the V_S plays an important role in the observed RS effect in MoS_2 memristors.

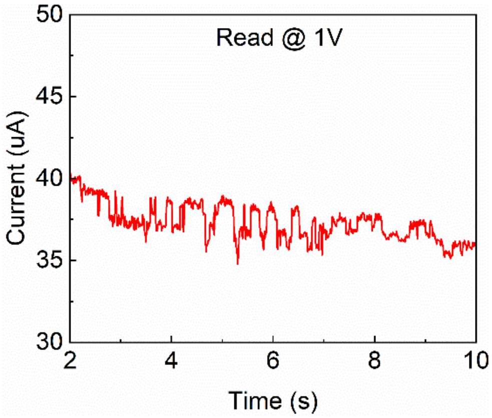

Supplementary Figure S8| Random telegraph noise (RTN) fluctuations of MoS_2 memristor. The RTN trace of MoS_2 memristor at HRS state was measured under a constant voltage of 1V applied on the top electrode. The RTN clearly shows two discrete conductance states, originating from the stochastic capture and emission of electrons at the defect sites near the conduction path.[1] In our devices, the V_S defects are responsible for random trapping/de-trapping of tunneling electrons.

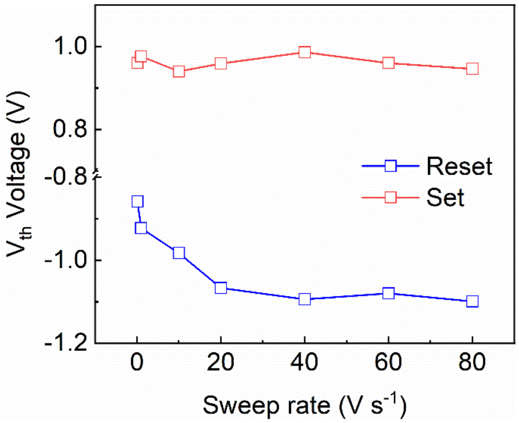

Supplementary Figure S9 | Study of the dependence of threshold voltage on the sweep rate. The threshold voltage exhibits a strong dependence on the sweep rates, implying that a nanoionics transport mechanism dominates the memory effect of solution-processed MoS_2 memristors, where slower sweep rates afford more time for V_S diffusion, resulting in the reduction in threshold voltages.

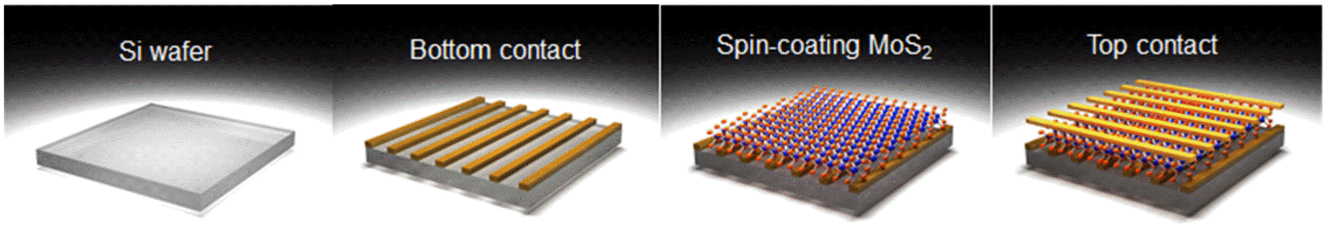

Supplementary Figure S10 | Schematic diagram showing the fabrication process of the solution-processed MoS_2 memristor. The MoS_2 nanosheets are assembled by direct spin-coating onto the pre-patterned bottom contact (Ti/Pt) at room temperature. This enables the facile fabrication on rigid and deformable substrate for emerging applications including flexible electronics and 3D monolithic circuits. After that, Ti/Pt electrodes are deposited on top of the MoS_2 thin film by e-beam evaporation to complete the device fabrication.

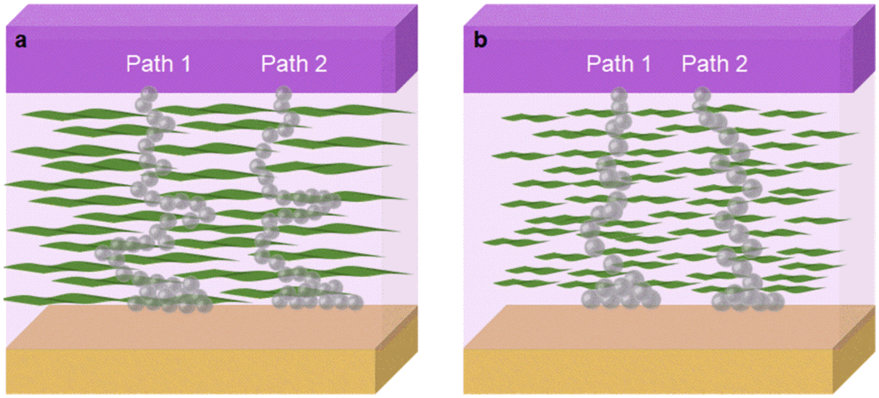

Supplementary Figure S11| Sulfur vacancy percolation path at different nanosheet size. a. MoS_2 memristor with large nanosheet size. **b.** MoS_2 memristor with small nanosheet size. To be noted, the grey spheres represent V_S. The cycle-to-cycle variation depends on the uniformity of V_S percolation path in our MoS_2 memristors. Large nanosheet size has much higher randomness in the V_S percolation path, causing switching uniformity deterioration.

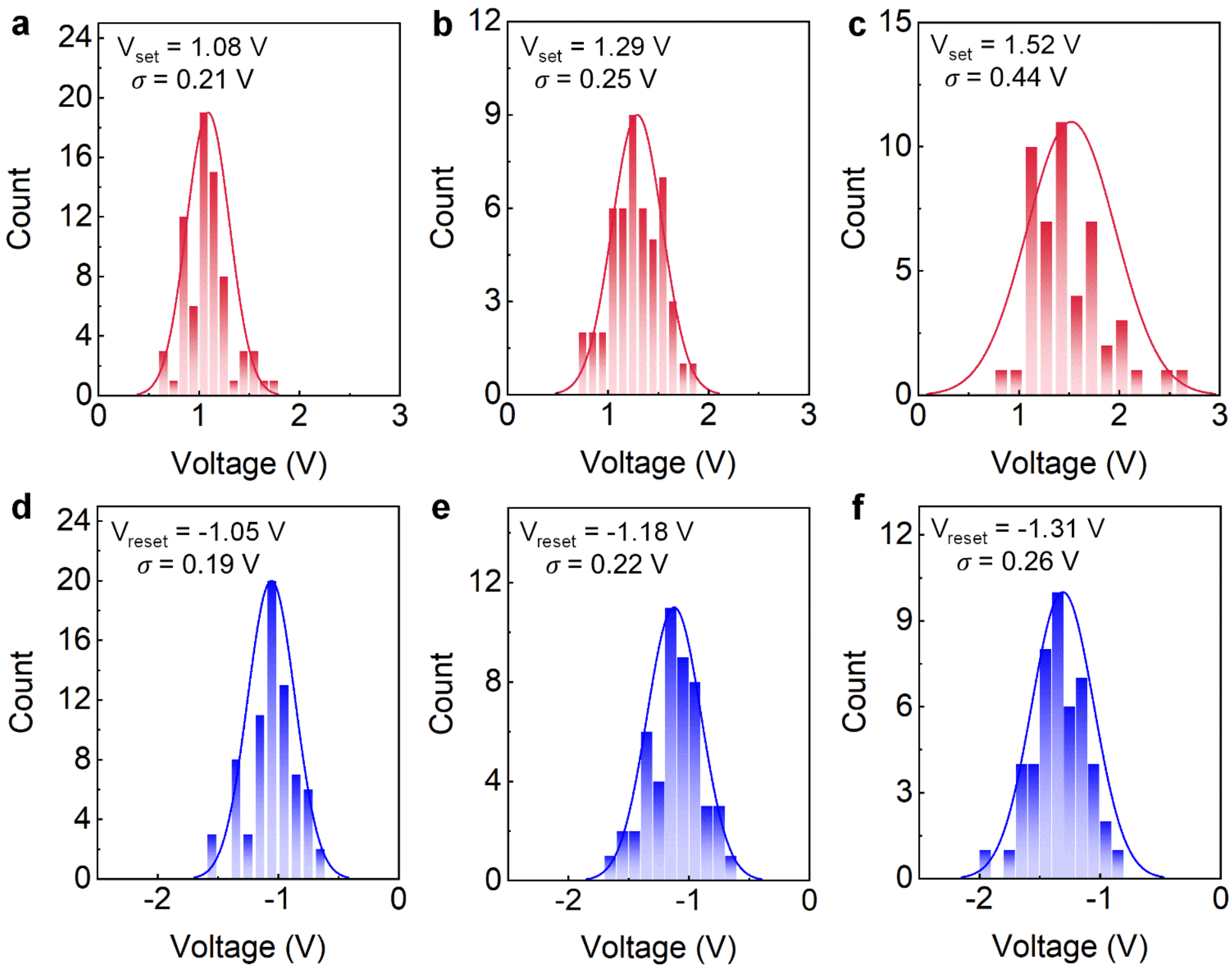

Supplementary Figure S12 | Statistical analysis of the switching voltages as a function of MoS_2 nanosheets size. Histogram of the set voltage (V_{set}) and reset voltages (V_{reset}) from MoS_2 suspension A (**a, d**), B (**b, e**) and C (**c, f**), respectively. To be noted, V_{reset} is defined as the voltage at the maximum current under negative bias. V_{Set} is defined as the voltage where the current abruptly increases under positive bias. The statistical analysis of the standard deviation (σ) and average values of switching voltages of 50 devices in each batch reveal the improved switching uniformity and feasibility when shrinking the nanosheet size. Specifically, the RS happens at higher voltages of 1.52 V (set) and -1.31 V (reset) with a larger σ of 0.44 V and 0.26 V for MoS_2 suspension C. In contrast, a tighter distribution in V_{set} and V_{reset} together with smaller switching voltages has been achieved when

MoS_2 suspension A is used. This confirms the strong correlation between the flake geometry and the corresponding RS characteristics.

Note I: Discussion on the density function calculation (DFT) of the V_S diffusion

To figure out the diffusion of V_S crossing layers, we build the three-layer MoS_2 nanoflakes with different widths and the calculated diffusion energy barriers are shown in Figure S13. Given the van der Waals gap existed in the MoS_2 adjacent layers, defects can drift only in fixed positions, namely the defective edges. It can be seen that the V_S diffusion energy barrier is 1.49 eV when the width of nanoflake is 19.4 Å. The diffusion barrier is slightly decreased to 1.08 eV when the width of nanoflake decreases to 11.0 Å. When the size of nanoflake is further reduced to 8.4 Å, the diffusion barrier of V_S is dramatically reduced to 0.75 eV. Based on our simulation results, we consider that when the flake size is smaller, the diffusion barrier of V_S is lower. The mild diffusion barrier would be beneficial to the switch function in MoS_2 memristor. For comparison, we calculated the diffusion barrier of V_S perpendicular to the basal plane of the perfect crystalline bilayer MoS_2 as shown in Figure S13e, and the calculated diffusion barrier is 4.35 eV, which is much higher than that in nanoflake of 0.75 eV. Therefore, we suggest the epitaxial MoS_2 single crystal is not suitable for memristor device due to ultrahigh diffusion barrier of V_S. Our predictions are consistent with the reported experimental results.[2] The calculated diffusion barrier of 0.75 eV for V_S is slightly higher than that our previously predicted value of 0.61 eV in polycrystalline monolayer MoS_2. In which, the V_S diffusion accompanied with the glide of (4|6) dislocation, and taken place in interlayer polycrystalline monolayer MoS_2.[3]

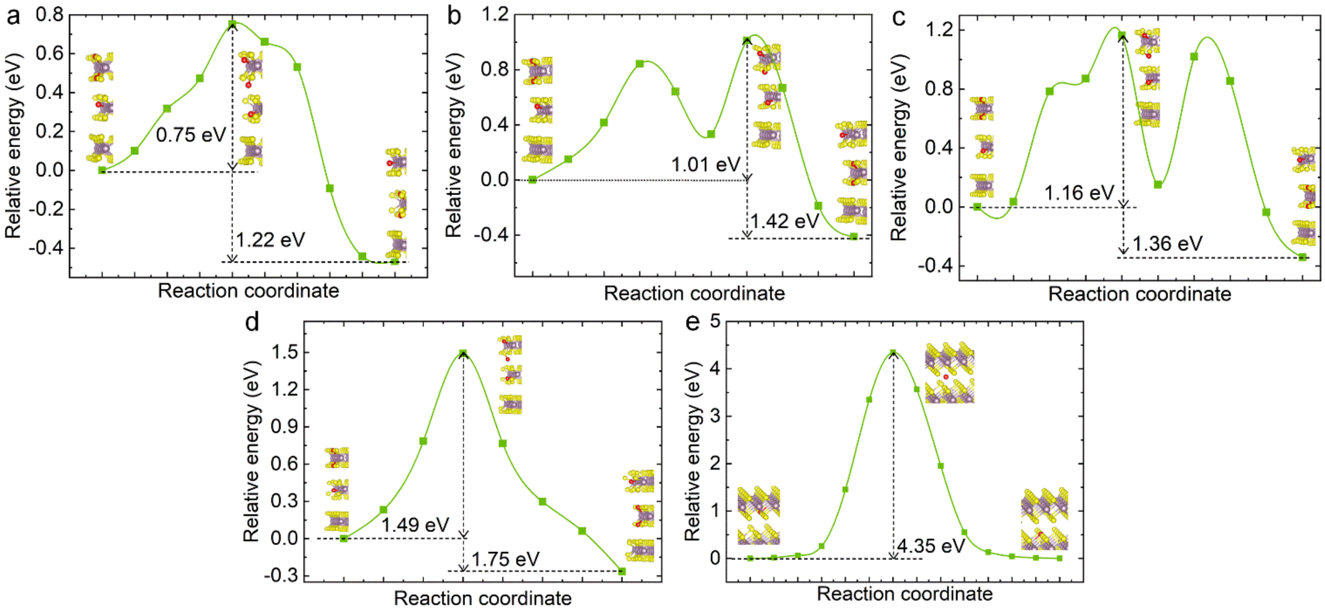

Supplementary Figure S13 | Energy landscape for V_S interlayer diffusion. Three-layer MoS_2 with width of **a.** 0.84 nm, **b.** 1.10 nm, **c.** 1.38 nm and **d.** 1.94 nm. **e**. The perfect two-layer MoS_2

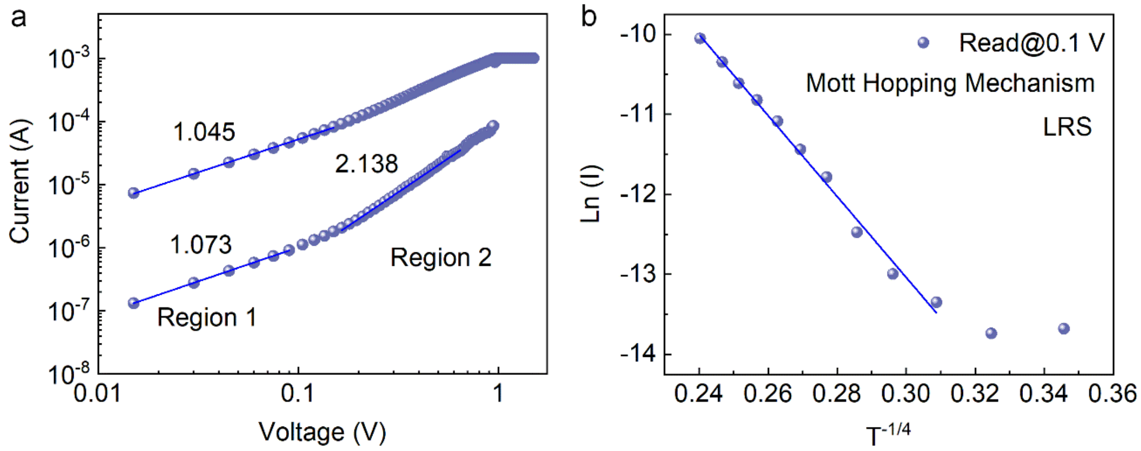

Supplementary Figure S14 | Electrical investigation of the charge transport mechanism. a. Double-logarithmic plots of the *I-V* curve of MoS_2 memristor. **b.** The relationship of conductivity ln *I* versus temperature $T^{-1/4}$ in LRS state. The read voltage is 0.1 V. As shown in the double-logarithmic plots of the *I-V* curve, the LRS state shows an Ohmic conduction behavior with a slope close to 1, which is caused by the formation of conductive filaments.[4] The log *I-V* plot of HRS in the low voltage region (<0.2 V) obeys the Ohmic conduction behavior and gradually

changes to a square dependence ($|I| \propto |V|^2$). This behavior is qualitatively interpreted to follow the shallow trap-associated space-charge-limited conduction (SCLC) theory, expressed by $I(\mathrm{V})=aV+bV^2$. At low voltage, corresponding to Ohm's law, only a few electrons can be generated due to thermal excitation and get excited to the conduction band from the valance band or the impurity level in this voltage range. When the applied voltage increases, the conduction becomes space-charge-limited and follows a square dependence. Once the conductive filament forms, a transition from HRS to LRS occurs and the log I-V plot follows the ohmic conduction behavior.

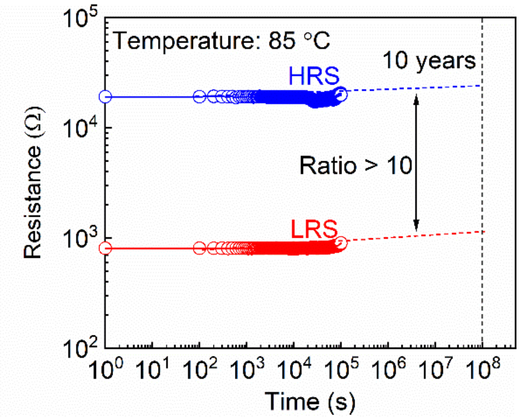

Supplementary Figure S15 | Data retention performance of the MoS_2 memristor. The MoS_2 memristor at HRS and LRS state was measured under 0.1 V bias (T = 358 K). The extrapolation method is employed to give a long-term (10-year) prediction result.

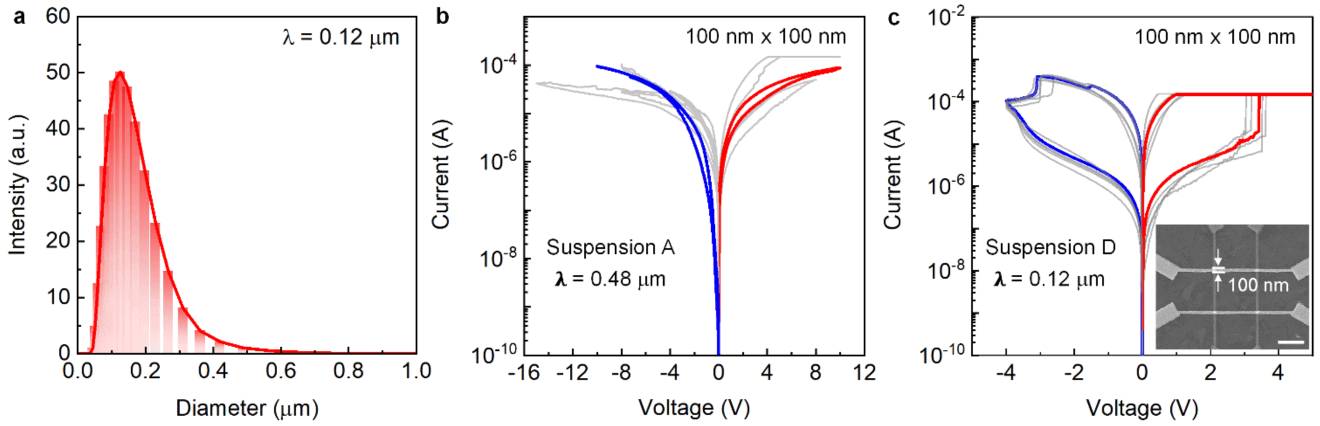

Supplementary Figure S16 | Scaled MoS_2 memristor. a. The lateral flake size distribution of MoS_2 suspension D. Scaled MoS_2 RRAM (100 nm×100 nm) made from (**b**) MoS_2 suspension A (**λ** = 0.48 μm) and (**c**) suspension D (**λ** = 0.12 μm). **λ** is the average nanosheet size. Owing to the edge switching effect, the scaled device shows negligible RS effect when large nanosheet size was used as shown in Figure S16b. There are not enough edge sites or defects within the electrode region to facilitate the resistive switching. However, when MoS_2 nanosheet size reduces to around 0.12 um, uniform bipolar resistive switching behavior are observed in the scaled devices (Figure S16c). It verifies the scalability of the proposed concept since the MoS_2 nanosheet size is also tunable within a wide range.

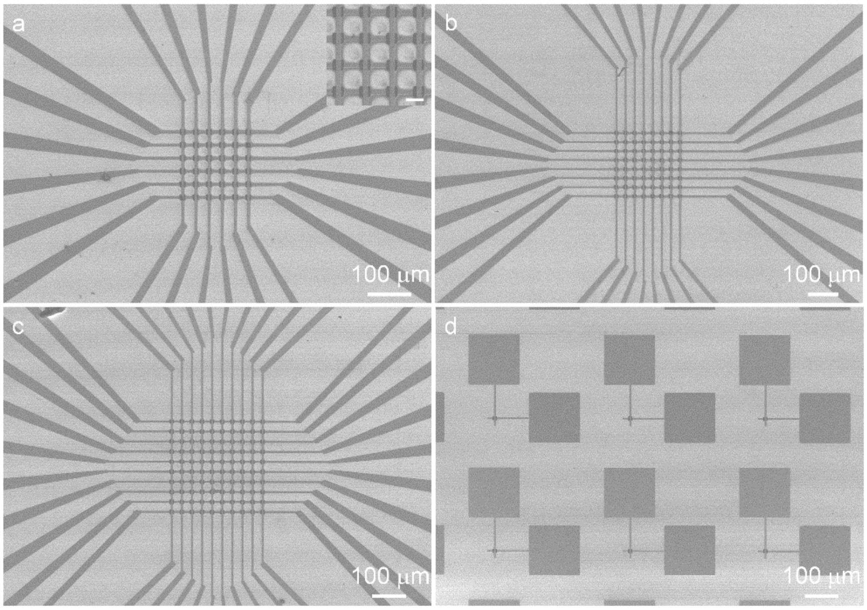

Supplementary Figure S17 | Scanning electron microscopy (SEM) image of the MoS_2 memristor crossbar arrays selected from the wafer-scale MoS_2 memristor crossbar arrays. a. 6×6 arrays, **b.** 8×8 array, **c.** 10×10 array and **d.** individual crossbar. The scalebar in the inset is 20 μm.

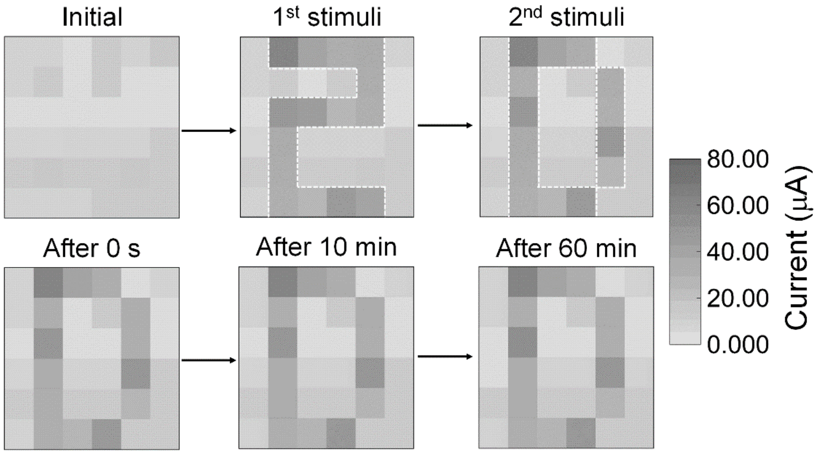

Supplementary Figure S18| Conductance maps of the 6×6 MoS_2 memristor array. A number '2' and Alphabet 'D' are programmed in succession by switching selected cells into the LRS. Pulse voltage of 1.5 V (set) and -2.0 V (reset) with pulse width of 1 us are used in the programming. The current was measured with pulse amplitude of 0.2 V at pulse width of 1 us.

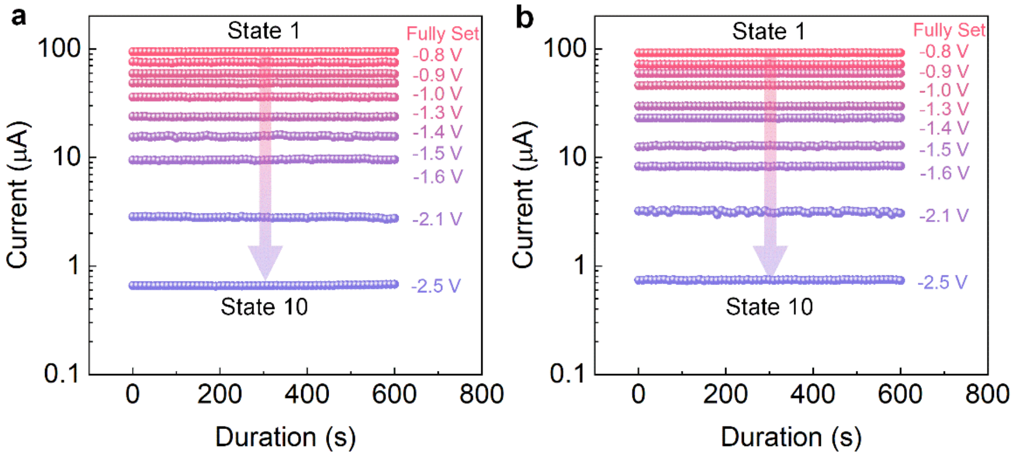

Supplementary Figure S19 | Repeatable programming of multiple memory states in MoS_2 memristor. *I-t* measured at 0.2 V for 10 memory states of (**a**) initially programmed and (**b**) re-programmed MoS_2 memristor. "Fully set" refers to the memory state when device was fully set to LRS states by sweeping a positive bias to 2 V with compliance of 1 mA. After the MoS_2 memristor was programmed from State 1 to State 10, it is fully set back to State 1 with 2V bias under 1 mA compliance (reprogram process). The value beside the curve refers to the reset voltage for each memory state. The repeatable memory states in the same device indicates those memory states are reproducible and programmable.

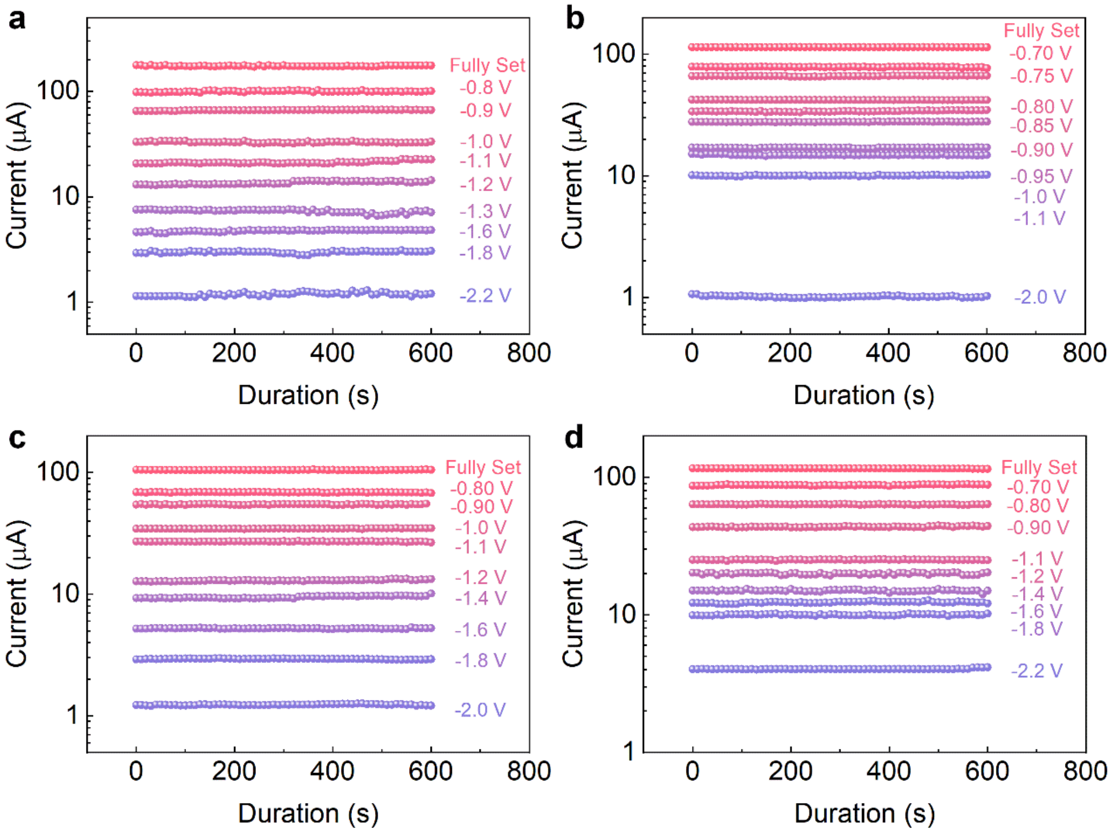

Supplementary Figure S20 | Multiple memory states reproducible in different MoS_2 memristors. *I-t* measured at 0.2 V for 10 programmed memory states on four MoS_2 RRAM devices randomly selected on the same batch (**a**) device 1, (**b**) device 2, (**c**) device 3 and (**d**) device 4. The value beside the curve refers to the reset voltage for each memory state.

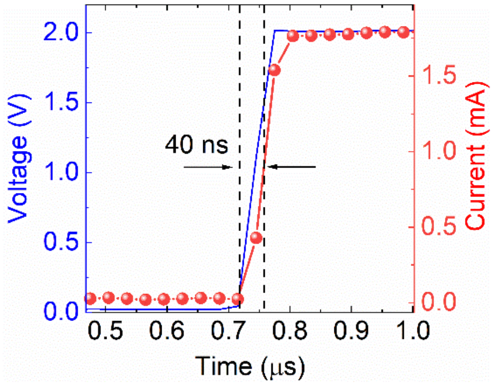

Supplementary Figure S21| Evaluation of the MoS_2 memristor switch time. Switch time characterization with AC pulse of 2.0 V amplitude and 500 ns pulse width.

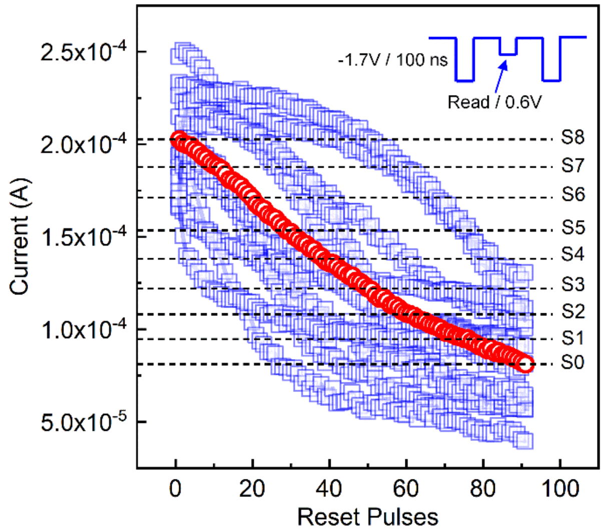

Supplementary Figure S22 | Conductance modulation of MoS_2 memristors with device-to-device variability. In total, 20 memristors have been measured with 90 identical reset pulses. The conductance curves are divided into 9 states based on the demonstrated device-to-device variability.

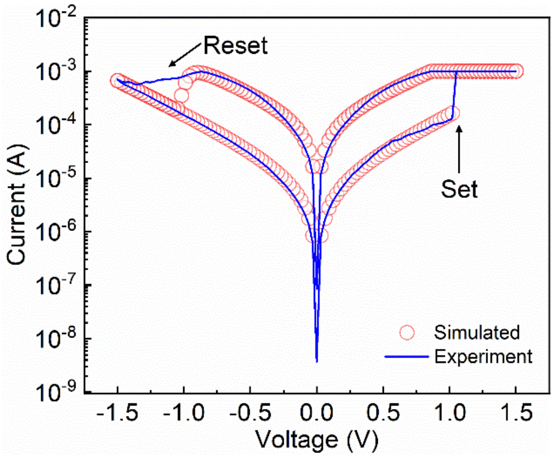

Supplementary Figure S23 | Calibration of experimental data with the Hysteron-based model. Comparison of developed spice model with experimental data shows good correlation.

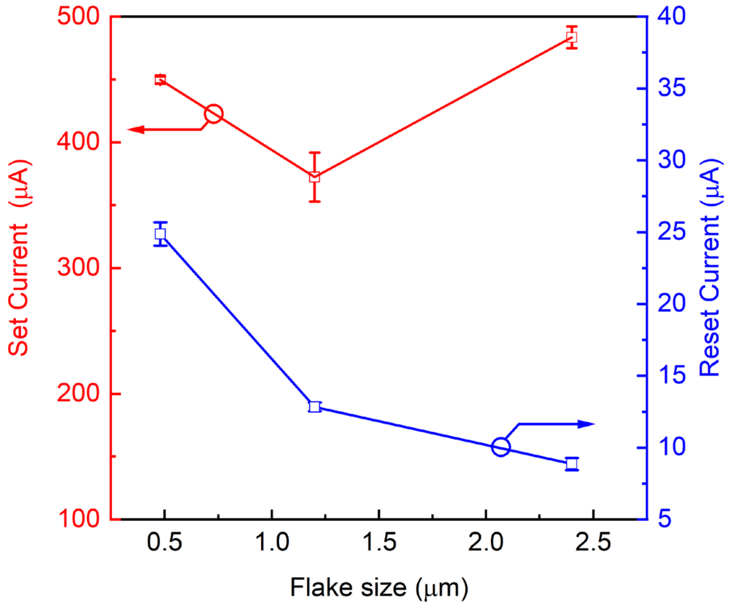

Supplementary Figure S24 | HRS and LRS current distribution of MoS_2 RRAM as a function of nanosheet size. The current of HRS and LRS current was read at 0.6 V. Data are presented as mean ± the set/reset current distribution.

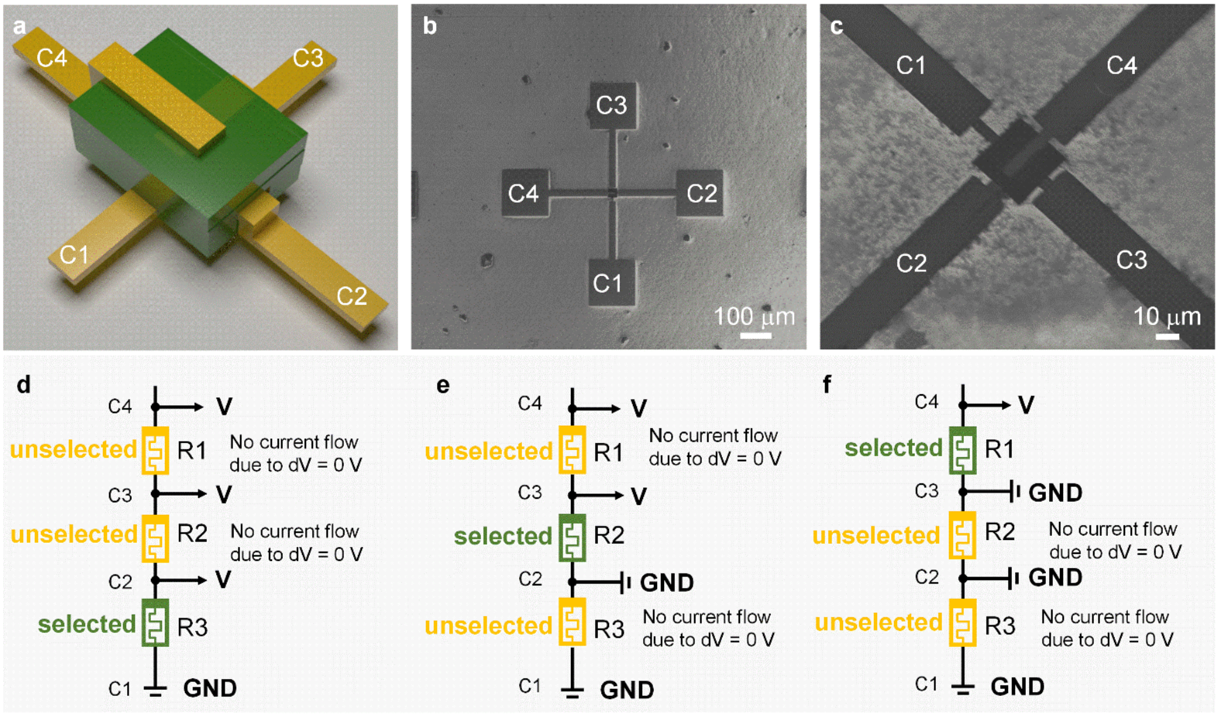

Supplementary Figure S25 | 3D stacked MoS_2 memristors. a. The schematic diagram and corresponding SEM images (b-c) of the 3D MoS_2 memristor stacks. **d-f.** Equivalent circuits representing the voltage scheme used for addressing selected memristor in the bottom, middle and top layer, respectively. C1, C2, C3 and C4 denote the metal contacts for each layer. The 3D MoS_2 RRAM stacks consist of C1/MoS_2/C2 (bottom layer), C2/MoS_2/C3 (middle layer) and C3/MoS_2/C4 (top layer), respectively. Each layer of the MoS_2 RRAM can be accessible and programmable independently in which MoS_2 serves as the separation layer. For example, the bottom RRAM is selected when a voltage bias of V is applied on electrode C2 with C1 grounded. C3, and C4 are given the same voltage as C2, thus no current flow through C3 and C4 due to zero voltage potential drops.

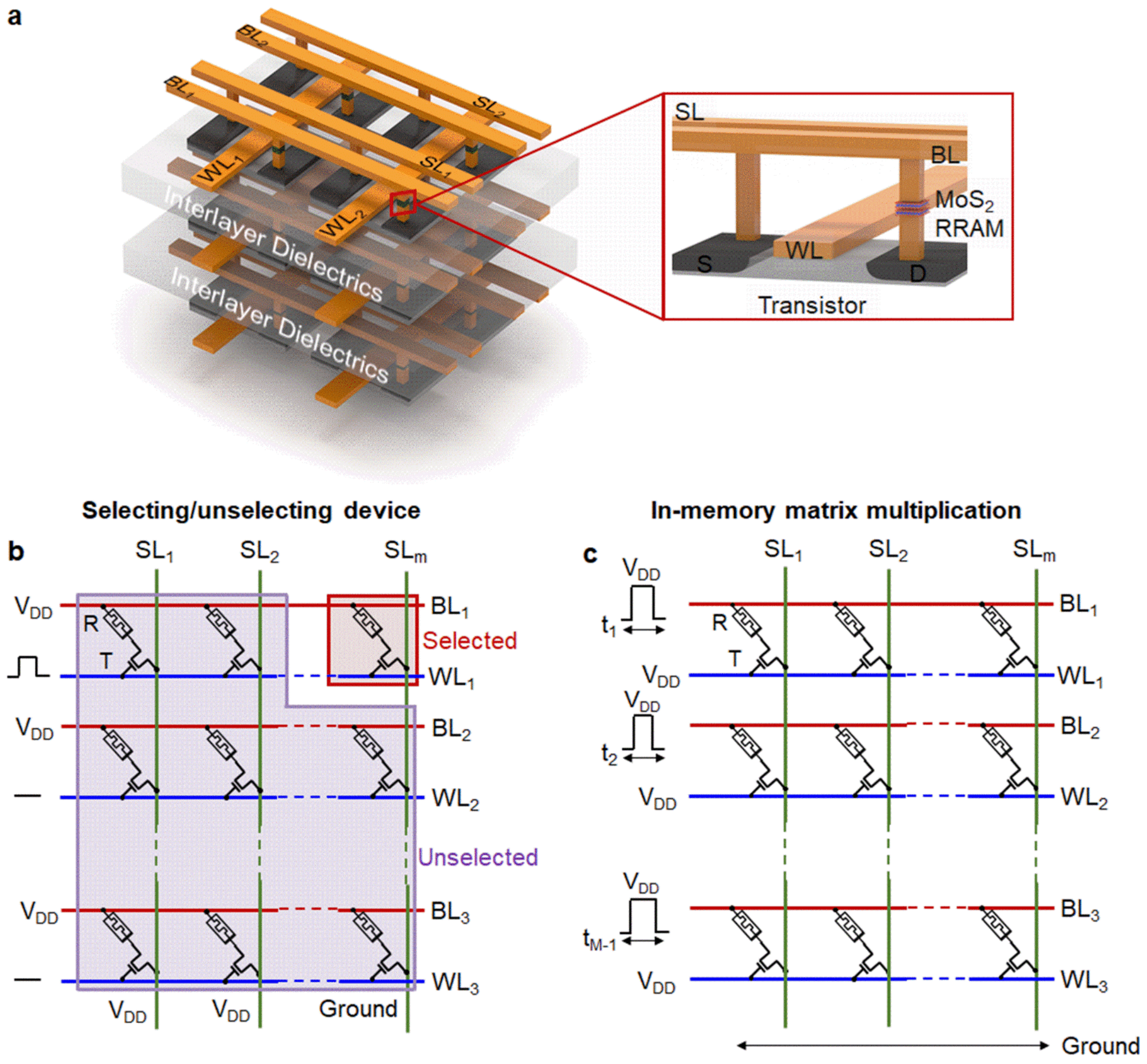

Supplementary Figure S26 | Illustration of monolithic 3D integration of one transistor one RRAM (1T1R) array. a. The schematic diagram of a three-layered 1T1R M3D arrays where MoS_2 memristor is in series with a selection transistor to eliminate the sneak path current. The inset showing the detailed structure of the memory cell. SL, BL and WL represent source line, bit line and world line. The gates of transistor and top electrodes of RRAM are connected by the WL and BL, respectively. The source of the transistor is connected to the SL. **b.** Equivalent circuits showing the voltage scheme used for addressing selected RRAM. WL serves as the selecting

line. Signal inputs are applied to the BL, while signal outputs are collected at SL. **c.** Equivalent circuits showing the voltage scheme used for performing in-memory matrix multiplication within the RRAM arrays.

To select one cell, different WL voltages are used for SET and RESET (Fig. S26b). For example, for SET, a small voltage is applied on WL to turn on the selection transistor, while BL voltage is applied to set the state. For RESET, a large voltage is applied on WL to turn on the selection transistor while compensating the voltage drop on the RRAM cell. Meanwhile, SL voltage is applied to reverse the current to reset the RS state in a typical bipolar RRAM. For the implementation of in-memory matrix multiplication, kernel elements are stored within the RRAMs as conductance levels. Information is stored in the time period of the voltage pulses applied at BL. Since input pulses are applied to all the RRAMs in the array simultaneously, V_{DD} is applied at the WL of all transistors. Signal outputs are collected at SL (Fig. S26c).

Note II: Details on the CNN simulations

To test the usability of the solution-processed MoS_2 array for neural networks, we simulated a 3-layer CNN with 1 convolution layer and 2 fully connected layers for the classification of MNIST handwritten digit database.[5] Details of the layers and the filter sizes is given in the table below:

Supplementary Table I Simulated neural networks

NN	Layer Weights	This Work	GPU
1 FC+<br>1 SM	FC: 30×196<br>SM: 10×30	95.36	95.69

1 CN+<br>1 FC+<br>1 SM	CN: (7×7, 5, 1)<br>FC: 30×605<br>SM: 10×30	98.02	98.24

FC: *Fully Connected layer;* ***SM:*** *Softmax Layer;* ***CN:*** *Convolution Layer+ Max-pool Layer;* ***CA:*** *Classification Accuracy;* ***NN:*** *Neural Network;* ***#Epochs****=100*

The unique aspects for our CNN implementation, which includes conductance discretization based on variability and our floating-point mapping techniques, effectively combat device variability to reduce the system output error. We have previously implemented similar algorithm for oxide-based RRAM,[6,7] and in this work, we successfully demonstrate system output error reduction with 2D materials in a memory array simulation. The system evaluation is based on variations captured through multi-devices characterizations, rather than single device data (Supplementary Fig. S22).

We discretize the RRAM conductance curve into a finite number of states determined based on the observed device variability. The kernel elements are mapped onto those discrete states and stored within the RRAMs for processing. We show in our previous works that the conductance discretization based on variability and our mapping techniques effectively combat device variability to reduce the system output error. Also, we employ pulse width modulation to execute computations within the RRAMs to reduce the power and area consumed by the periphery. In our current implementation, we divide the RRAM conductance into 16 discrete states and use a 3-bit input image/ 4-bit kernel resolution to execute the CNN within RRAMs. The high-accuracy computations that we perform here using low resolution reduce system power/area compared to other works. A detailed description of our methodology, along with its performance analysis, has been provided in our previous works.[6,7] In our execution, we modeled the RRAM behavior based on the conductance curves of 20 devices over multiple cycles. We derived the variance/mean of the measured data and used this to run our simulations. We modeled

the current variation at each discrete state as a gaussian function about the mean and derived the conductance at random from this function at every iteration. Thus, the simulations performed in our work account for device irregularities such as limited conductance range and variability to provide an accurate estimate of the output errors. The sample conductance curves of 20 RRAMs (cell size 5×5 μm^2) used in this work, along with their discretization into 9 states, is provided in the Figure S22. For system analysis, a hysteron-based compact model, developed by Lehtonen et.al.,[8] has been calibrated to our MoS_2 RRAM in Figure S23.

CNN performance depends on the non-linearity, conductance range, and variability exhibited by the RRAM devices. Figure S22 illustrates that our RRAM devices demonstrate a 2× linear conductance change over 90 reset pulses. Hence, we determined the memory window and the confidence intervals for the conductance spread of devices fabricated with different suspensions using the bootstrap method in **R**. We documented the results in Figure S24. The figure delineates that the memory window increases progressively with nanosheet size. While the conductance spread does not follow a pattern, it is <10% of the mean for all nanosheet sizes considered. Thus, our CNN simulations, which we performed with a σ/μ of 0.35 on devices fabricated with nanosheet size of 0.48 μm, account for the worst-case scenario. Furthermore, as explained previously, our in-memory compute methodology accounts for device variability issues, thereby preventing accuracy degradation. Hence, the CNN classification accuracy would remain unaltered for different nanosheet sizes.

Supplementary Table II Benchmarking of our solution-processed MoS_2 RRAM devices with reported 2D materials-based RRAM and conventional oxide-based RRAM.

Active layer	$V_{SET/RESET}$	Forming voltage	Compliance current	Digital/ Analog	Conductance states	Endurance	Array size	Retention
Colloidal MoS_2[9]	5.5/-0.5V	Form-free	0.1 mA	digital	2	100	10×10	10^4 s
Printed MoO_X-MoS_2[10]	0.15/-0.1V	Form-free	0.5 mA	digital	2	50	single	8000 s
Aerojet printed MoS_2[11]	0.18/-0.3V	Form-free	1 mA	digital	2	200	single	10^5 s
Graphene/MoS_2/Graphene[12]	1.2/2.2V	1.5V	0.7 mA	digital	2	10^7	4×4	10^5 s
Black phosphorus[13]	3/-2.8V	Form-free	1 mA	digital	2	-	single	10^4 s
Graphene oxide[14]	2.5/-2.5V	Form-free	0.05 mA	analog	-	100	5×5	10^5 s
CVD h-BN[15]	2.65/-1.85V	3~8.0V	1 μA	analog	26	8000	10×10	-
h-BN/Graphene/h-BN[16]	4.0/3.8V	5.0V	0.1 mA	digital	2	10^6	12×12	10^6 s
Pd/HfO_x/TiN[17]	2.2/-2.2 V	5.25 V	0.1 mA	analog	-	$>10^8$	single	10^4 s
TaO_x/Ta_2O_5[18]	-1.0/1.5 V	-	0.1 mA	analog	-	10^7	single	10^8 s
**This work**	**0.65/-0.9V**	**Form-free**	**1 mA**	analog	**50**	**10^7**	**10×10**	10^8 s

Supplementary References

1 Ielmini, D., Nardi, F. & Cagli, C. Resistance-dependent amplitude of random telegraph-signal noise in resistive switching memories. *Appl. Phys. Lett.* **96**, 053503 (2010).

2 Sangwan, V. K. *et al.* Multi-terminal memtransistors from polycrystalline monolayer molybdenum disulfide. *Nature* **554**, 500-504 (2018).

3 Yu, Z. G., Zhang, Y.-W. & Yakobson, B. I. An anomalous formation pathway for dislocation-sulfur vacancy complexes in polycrystalline monolayer MoS_2. *Nano lett.* **15**, 6855-6861 (2015).

4 Chiu, F.-C. A review on conduction mechanisms in dielectric films. *Adv. Mater. Sci. Eng.* **2014**, 578168 (2014).

5 LeCun, Y., Cortes, C. & Burges, C. J. Minist handwritten digit database. *AT&T Labs.* **2**, http://yann.lecun.com/exdb/mnist (2010).

6 Veluri, H., Li, Y., Niu, J. X., Zamburg, E. & Thean, A. V. Y. High Throughput, Area-Efficient, and Variation-Tolerant 3D In-memory Compute System for Deep Convolutional Neural Networks. *IEEE Internet Things J.*, 1-1, **8**, 9219-9232 (2021).

7 Veluri, H., Chand, U., Li, Y., Tang, B. & Thean, A. V.-Y. A Low-Power DNN Accelerator Enabled by a Novel Staircase RRAM Array. *IEEE Trans. Neural Netw. Learn. Syst.* DOI: 10.1109/TNNLS.2021.3118451 (2021).

8 Lehtonen, E. & Laiho, M. in *2010 12th International Workshop on Cellular Nanoscale Networks and their Applications (CNNA 2010).* 1-4 (IEEE).

9 Son, D. *et al.* Colloidal synthesis of uniform-sized molybdenum disulfide nanosheets for wafer-scale flexible nonvolatile memory. *Adv. Mater.* **28**, 9326-9332 (2016).

10 Bessonov, A. A. *et al.* Layered memristive and memcapacitive switches for printable electronics. *Nat. Mater.* **14**, 199-204 (2015).

11 Feng, X. *et al.* A Fully Printed Flexible MoS_2 Memristive Artificial Synapse with Femtojoule Switching Energy. *Adv. Electron. Mater.* **5**, 1900740 (2019).

12 Wang, M. *et al.* Robust memristors based on layered two-dimensional materials. *Nat. Electron.* **1**, 130-136 (2018).

13 Han, S. T. *et al.* Black phosphorus quantum dots with tunable memory properties and multilevel resistive switching characteristics. *Adv. Sci.* **4**, 1600435 (2017).

14 Jeong, H. Y. *et al.* Graphene oxide thin films for flexible nonvolatile memory applications. *Nano Lett.* **10**, 4381-4386 (2010).

15 Chen, S. *et al.* Wafer-scale integration of two-dimensional materials in high-density memristive crossbar arrays for artificial neural networks. *Nat. Electron.* **3**, 638-645 (2020).

16 Sun, L. *et al.* Self-selective van der Waals heterostructures for large scale memory array. *Nat. Commun.* **10**, 1-7 (2019).

17 Wu, Q. *et al.* Improvement of durability and switching speed by incorporating nanocrystals in the HfOx based resistive random access memory devices. *Appl. Phys. Lett.* **113**, 023105 (2018).

18 Lee, S. R. *et al.* Multi-level switching of triple-layered TaO_x RRAM with excellent reliability for storage class memory. in *2012 Symposium on VLSI Technology (VLSIT).* 71-72 (IEEE).
